# Radiation induced angiosarcoma of the breast: outcomes from a retrospective case series

**DOI:** 10.1186/s13569-017-0081-7

**Published:** 2017-08-07

**Authors:** R. B. Cohen-Hallaleh, H. G. Smith, R. C. Smith, G. F. Stamp, O. Al-Muderis, K. Thway, A. Miah, K. Khabra, I. Judson, R. Jones, C. Benson, A. J. Hayes

**Affiliations:** The Sarcoma Unit, The Royal Marsden Hospital NHS Foundation Trust, London, UK

**Keywords:** Radiation, Angiosarcoma, Breast

## Abstract

**Background:**

Radiation induced angiosarcoma (RIAS) of the breast is a rare and aggressive complication of radiotherapy. Due to the rarity of this disease, much of the evidence for its management is based on case reports or small retrospective series. We sought to describe the management and outcomes of RIAS in a large single-institution series.

**Methods:**

All patients diagnosed with RIAS between January 2000 and January 2014 were identified from an institutional database.

**Results:**

A total of 49 patients were identified. Median age at diagnosis was 72 years (range 51–93). Median time from completion of radiotherapy to diagnosis of RIAS was 7.5 years. Median tumour size at presentation was 5.0 cm (1.5–19.0). The majority of patients presented with localised disease (47, 95.9%). Of these, 35 (74.5%) were suitable for surgery and underwent surgery with curative intent. Twelve patients presented with localised irresectable disease. Of these, 7 received systemic chemotherapy, with a sufficient response to facilitate surgery in 3 patients. Following potentially curative surgery, 2-year local recurrence-free was 55.2%. Survival was significantly prolonged in patients presenting with resectable disease (2-year overall survival 71.1% vs 33.3%, p < 0.001). Tumour size >5 cm was prognostic of distant metastases-free survival and overall survival.

**Conclusion:**

RIAS are rare, aggressive soft-tissue lesions with limited treatment options and high-rates of both local and systemic relapse.

## Background

Radiation-induced angiosarcoma of the breast (RIAS) is a rare and late complication of radiotherapy for breast cancer. In those patients undergoing breast conserving surgery with adjuvant radiotherapy, the estimated incidence of RIAS is 0.05–0.3% [1–4]. Although still rare, the incidence of RIAS appears to be increasing, perhaps reflecting the long latency period for the development of these tumours following the widespread adoption of adjuvant radiotherapy for breast cancer. In a large population-based cohort study, a history of prior radiotherapy as a treatment for breast cancer was associated with 26-fold increase in the risk of developing angiosarcoma when compared with non-irradiated controls [5]. The prognosis for patients with RIAS remains poor, with 5-year overall survival rates ranging from 27 to 48% [2]. Surgery, in the form of wide excision or mastectomy, is the mainstay of management in localised disease. Some studies have reported an association between R0 margins and improved survival, although this was not demonstrated to be independent of other biological factors such as tumour size [6, 7]. Although there is some evidence that neoadjuvant chemotherapy may improve outcomes in angiosarcoma, the rarity of this condition limits such evidence to case reports or small retrospective series [8–12]. The purpose of this study was to describe the management and outcomes of patients presenting with RIAS of the breast within a large single-institution case series.

## Methods

All patients treated with a diagnosis of RIAS at The Royal Marsden Hospital between January 2000 and January 2014 were identified from a prospectively maintained database. Ethical approval was obtained from an institutional review board. RIAS was defined as a histologically proven diagnosis of angiosarcoma occurring in a patient with a history of irradiation of the surgical field following breast-conserving surgery for breast cancer.

### Operative strategy

Patients either underwent their initial surgical management at The Royal Marsden Hospital or were referred following an initial resection elsewhere. All patients undergoing surgery at The Royal Marsden Hospital were discussed at a sarcoma multidisciplinary meeting pre-operatively. Patients were classified as having resectable disease if pre-operative assessment indicated that a 2 cm or greater negative margin could be achieved by surgery with or without plastic surgical reconstruction in the form of a single pedicled or free myocutaneous flap. If the desired negative margins would require more extensive reconstruction, such as with extensive resurfacing by large skin grafting, the patient was classified as having irresectable disease. Rapidly progressive disease, where disease volume increased over a time span of 2–3 weeks from being suitable for mastectomy alone or in combination with a pedicled flap to requiring more extensive reconstruction, was also judged irresectable in oncological terms. Pre-operative 4-quadrant punch biopsies were performed to confirm that the planned surgical margins were not involved by microscopically occult disease. Macroscopically complete resection was judged by the operating surgeon. Histologically, the resection was classified as R0 (microscopically negative) if the negative margins were >1 mm circumferentially or R1 (microscopically positive) if tumour extended to or within 1 mm of the resection margin.

### Statistical analyses

The latency period to the development of RIAS was defined as the time from the date of completion of radiotherapy to the date of a histological diagnosis of RIAS. Local recurrence-free survival (LRFS) was defined as the time from histological diagnosis to the development of a local recurrence or last follow-up. Distant metastases-free survival (DMFS) was defined as the time from histological diagnosis to the development of distant metastases or last follow-up. Overall survival (OS) was defined as the time from histological diagnosis to the date of death or last follow-up. Survival curves were constructed using the Kaplan–Meier method and compared with the log-rank test. A univariate Cox regression analysis was used to investigate the following potential prognostic variables of LRFS, DMFS and OS: age; margin status; tumour size; treatment (surgery versus surgery and adjuvant chemotherapy). Potentially confounding factors (p < 0.1) were then combined in a multivariate analysis with forward stepwise combination methods. The results of these analyses are presented as hazard ratios (HR) with 95% confidence intervals (CI).

## Results

A total of 49 patients with a confirmed diagnosis of RIAS were identified during the study period. Patient demographics, primary breast cancer characteristics and treatment for primary breast cancer are outlined in Table 1. All patients were female, with a median age at diagnosis of RIAS of 72 years (range 51–93 years). The median time from completion of radiation therapy to the diagnosis of RIAS was 7.5 (range 1–26) years, with a median maximal tumour dimension of 5.0 cm (range 1.5–19.0 cm). None of the patients in this study had active breast cancer at the time of RIAS diagnosis, nor did any develop recurrent breast cancer during follow-up.

**Table 1:** Primary breast cancer characteristics and treatment in patients who developed RIAS

	N = 49 (%)
Median age at RIAS diagnosis (range)	72 (51–93)
Primary breast cancer histology	
Infiltrating ductal carcinoma	25 (51.0)
Infiltrating lobular carcinoma	3 (6.1)
Ductal carcinoma in situ	1 (2.0)
Unspecified	20 (40.8)
Surgery for primary breast cancer	
Wide local excision (WLE)	18 (36.7)
WLE and axillary lymph node dissection	29 (59.2)
Mastectomy	1 (2.0)
Mastectomy and LD flap reconstruction	1 (2.0)
Adjuvant therapies	
Chemotherapy	10 (20.4)
Endocrine therapy	36 (73.5)
Trastuzumab	3 (6.1)
Radiation dose (Gy) (range)	
Primary median	50 (40–54)
Boost median	12.5 (10–16)

*LD* latissimus dorsi

The majority of patients presented with localised disease (47 patients, 95.9%). Of these, 35 patients had resectable disease at presentation and underwent surgery with curative intent (74.5%). 25 patients (74.3%) underwent their initial operation at The Royal Marsden Hospital with 10 patients (25.7%) initially treated elsewhere. Of the 10 patients undergoing initial surgery elsewhere, 8 had a simple mastectomy, with 2 undergoing mastectomy with immediate plastic reconstruction with a pedicled flap (20.0%). Of the 25 patients undergoing initial surgery at The Royal Marsden Hospital, 9 had a simple mastectomy, with 16 undergoing a mastectomy with immediate plastic reconstruction (64.0%). A microscopically complete R0 resection was performed in 32 patients (91.4%). No further therapy was given to the majority of these patients, with 2 patients receiving adjuvant chemotherapy following surgery. The decision to give adjuvant chemotherapy was made based on the extent of disease, with 1 patient requiring both a pedicled flap and skin grafting to achieve macroscopic clearance and the other having a positive deep margin on the chest wall. Interestingly, neither of these patients developed local recurrence, though both subsequently relapsed systemically.

The remaining 12 patients with localised disease at presentation were considered to have irresectable disease (25.5%). Of these, 4 patients declined or were unfit for further intervention and received best supportive care (33.3%). Debulking surgery was performed in 1 patient for symptomatic palliation. This patient presented with large volume, fungating disease and a mastectomy was performed with no prospect of achieving clearance of all macroscopic skin changes. The remaining 7 patients were treated with systemic therapy, with 2 patients treated with doxorubicin and 5 patients receiving weekly paclitaxel. A sufficient response, downsizing the tumour to allow a potentially curative resection to be performed, was achieved in 3 patients. Local disease control was achieved in 2 of these patients, although both subsequently developed distant metastases.

Two patients presented with metastatic RIAS. The first patient presented with hepatic metastases and died following spontaneous haemorrhage from these lesions 5 months after diagnosis. The second patient with metastatic hilar and axillary lymphadenopathy responded well to paclitaxel chemotherapy and was disease free after 20 months follow-up.

### Outcomes

Of the 35 patients undergoing surgery for locally resectable disease, 18 developed a local recurrence (51.4%), 8 of whom presented with a synchronous systemic relapse (22.9%). 2-year LRFS was with 51.2% (95% CI 33.2–67.2). Of these 18 patients, 17 had microscopically negative margins following their initial surgery (94.4%). Resection margins in those patients who went on to develop local recurrence were significantly closer than those who did not (median clearance 1.0 cm vs 2.5 cm, p = 0.003, unpaired *t* test). All but 1 patient who developed local recurrence had less than 2 cm clearance. No difference in the proportion of patients developing local recurrence was noted in those undergoing reconstructive surgery and those closed primarily (44.4% vs 55.6%, p = 0.505, Fisher’s exact test). A further 7 patients developed isolated distant metastases, giving a systemic failure rate of 42.9%. 2-year DMFS was 67.3% (95% CI 48.6–80.5). At the time of writing, 20 patients had died (57.1%) with a 2-year OS of 71.1% (95% CI 52.9–83.3).

**Fig. 1:**
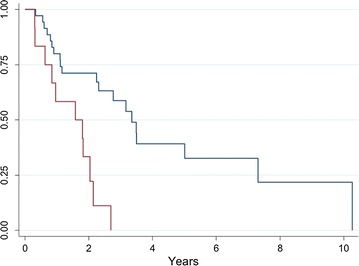
Overall survival from diagnosis of RIAS in patients with localised resectable (*blue*) and localised irrespectable (*red*) disease (p < 0.001, log-rank test)

Of the 12 patients with irresectable localised disease, 4 developed distant metastases (33.3%), with a 2-year DMFS of 57.3% (95% CI 21.6–81.7). At the time of writing, 11 of these patients had died (91.7%) with a 2-year OS of 33.3% (95% CI 10.3–58.8). Overall survival of patients with irresectable localised disease was significantly shorter than those with resectable disease (median OS 18 months vs 37 months, p < 0.001, log-rank test) (Fig. 1).

Univariate analyses were performed to identify prognostic factors of oncological outcomes in patients with resectable localised disease at presentation. The results are summarised in Tables 2 and 3. Tumour size and margin status were prognostic of DMFS on univariate analysis. However, with multivariate analysis, only tumour size remained prognostic of DMFS. Tumour size was also prognostic for OS, with no prognostic factors for LRFS identified.

**Table 2:** Univariate and multivariate Cox regressional analyses for prognostic factors of distant metastases-free survival in patients with localised resectable RIAS

Variable	Univariate		Multivariate	
	HR (95% CI)	p value	HR (95% CI)	p value
Age (years)				
<70	Reference	–	*	*
≥70	1.06 (0.98–1.15)	0.146	*	*
Positive margins				
No	Reference	–	*	*
Yes	4.20 (1.12–15.67)	0.033	*	*
Tumour size (cm)				
<5	Reference	–	Reference	–
≥5	5.70 (1.18–27.50)	0.030	5.70 (1.18–27.50)	0.030
Treatment				
Surgery	Reference	–	*	*
Surgery + chemotherapy	2.08 (0.46–9.51)	0.344	*	*

*Not included in model generated by forward stepwise combination

**Table 3:** Univariate Cox regressional analyses for prognostic factors of overall survival in patients with localised resectable RIAS

Variable	HR (95% CI)	p value
Age (years)		
<70	Reference	–
≥70	1.94 (0.77–4.91)	0.161
Positive margins		
No	Reference	–
Yes	1.17 (0.27–5.10)	0.837
Tumour size (cm)		
<5	Reference	–
≥5	5.18 (1.41–19.0)	0.013
Treatment		
Surgery	Reference	–
Surgery + chemotherapy	1.53 (0.35–6.72)	0.575

## Discussion

The widespread adoption of breast-conserving surgery and adjuvant radiotherapy in the management of primary breast cancer has been accompanied by a steady increase in the incidence of RIAS of the breast. RIAS is typically a late complication of adjuvant radiotherapy, with a median latency of 7.5 years in our institutional series, although there is considerable variation in the time to presentation, ranging from 1 to 26 years. These findings are consistent with those previously reported in the literature and due to the substantial variability in the latency of this disease, a high index of suspicion is warranted for any patient undergoing adjuvant radiotherapy in this context [1, 3, 13].

Surgery, in the form of mastectomy with or without plastic reconstruction, is the modality of choice in patients presenting with localised disease and achieved microscopically complete (R0) resection margins in more than 90% of patients in the current series. Despite this, the majority of patients developed local recurrence with a 2-year recurrence free survival of 55%. RIAS typically present as multifocal lesions and the propensity for this pathology to form microsatellite deposits may contribute to the difficulty in obtaining local control [3, 6, 14, 15]. The importance of performing a complete pathological resection has been stressed in the literature, although no standard guidelines regarding the recommended distance of clearance have been published [3, 16–18]. In the current series, those who developed local recurrence were found to have closer margins than those who did not, with only 1 of the 18 (5.6%) patients who recurred locally having more than 2 cm circumferential clearance. However, marginal status was not found to be independently prognostic of oncological outcomes in this series. This would suggest that the ability to achieve greater margins is dependent on other biological tumour factors that also determine outcome, such a size. Accordingly, no difference in local recurrence rates was noted between patients undergoing plastic reconstruction and those closed primarily. It is likely that the major determinant of outcome in RIAS is tumour biology and, although the initial surgery should aim for macroscopic clearance, it should be cautioned that achieving greater negative margins does not necessarily equate to improved patient outcomes.

**Fig. 2:**
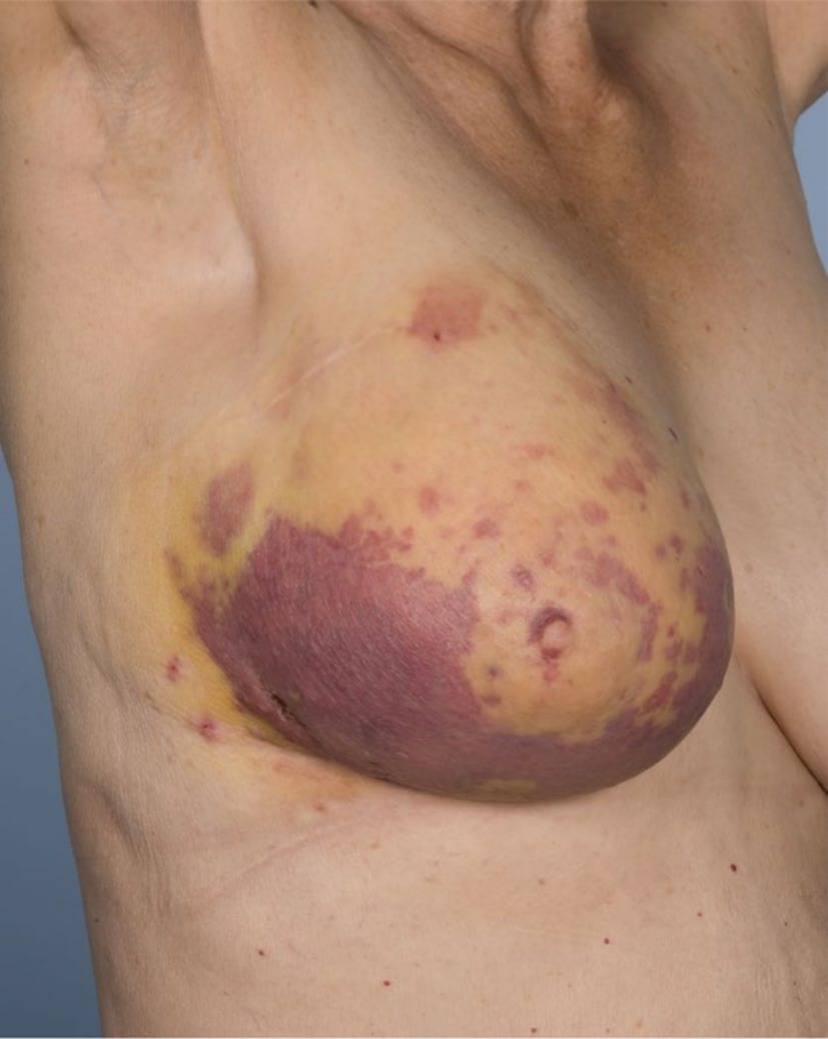
The typical appearances of radiation-induced angiosarcoma of the breast

Despite the majority of patients presenting with localised disease that was amenable to surgery, the rates of local and systemic relapse in RIAS are high. Tumour size was identified as the only independent prognostic factor of outcomes in this series, being associated with both DMFS and OS. A meta-analysis of patients with RIAS also identified tumour size as an important prognostic factor, being associated with LRFS and, alongside patient age, with OS [19]. As may be expected, poorer survival outcomes were noted in patients presenting with locally advanced disease unsuitable for surgical management in our series. These factors highlight the importance of early diagnosis in this patient group. Angiosarcomas often present insidiously with purple or red skin changes and may be easily mistaken for bruising or benign skin changes leading to delayed investigation and diagnosis (Fig. 2). Early detection and prompt referral may potentially reduce the number of patients presenting with irresectable disease and improve both local and distant disease control.

The role of peri-operative chemotherapy in the management of RIAS remains to be clarified. In the current series, of the 7 patients treated with neoadjuvant chemotherapy for localised irresectable disease, 3 patients achieved a sufficient response to facilitate surgery. Similar results were noted in the Phase II ANGIOTAX study, in which 30 patients with localised irresectable or metastatic angiosarcoma were treated with paclitaxel [20]. Five patients had partial responses, 3 of whom had localised irresectable lesions in the breast that were rendered resectable following treatment. On histopathological assessment of the resection specimens, 2 of these patients had achieved a complete histological response. The use of neo/adjuvant chemotherapy was also found to be associated with improved local disease control in a large retrospective series of patients with radiation-induced sarcomas of all sites, although not associated with improved rates of systemic relapse or survival [3]. Adjuvant chemotherapy was not found to produce a benefit in terms of local control or overall survival study of high-risk soft-tissue sarcomas treated with surgery and radiation [21]. As such, there is limited evidence to suggest that neo/adjuvant chemotherapy produces a survival benefit in RIAS, although it certainly may be of use as an induction therapy prior to surgery in those presenting with locally advanced disease and may offer patients effective disease palliation in addition. Targeted therapies may offer an alternative treatment in patients with progressive disease, with the tyrosine kinase inhibitor pazopanib demonstrating activity in both locally advanced and metastatic angiosarcoma [22].

RIAS are rare, aggressive soft-tissue lesions with limited treatment options and high-rates of both local and systemic relapse. Neoadjuvant chemotherapy may have a role in downsizing locally advanced disease although has no proven effect on survival.
